# The Benefits of a Surgery-First Approach in Promoting the Psychological Well-Being of Patients with Skeletal Anomalies and Temporomandibular Disorder

**DOI:** 10.3390/medicina61091598

**Published:** 2025-09-04

**Authors:** Simionescu Ana-Maria Andreea, Victor-Vlad Costan, Tinela Panaite, Anca Irina Gradinariu, Alina Elena Jehac, Adina Oana Armencia, Carina Balcos, Irina Nicoleta Zetu

**Affiliations:** Surgery Department, Faculty of Dental Medicine, “Grigore T. Popa” University of Medicine and Pharmacy, 700115 Iasi, Romaniavictor.costan@umfiasi.ro (V.-V.C.); tinela-panaite@umfiasi.ro (T.P.); anca.gradinariu@umfiasi.ro (A.I.G.); alina.jehac@umfiasi.ro (A.E.J.); adina.armencia@umfiasi.ro (A.O.A.); irina.zetu@umfiasi.ro (I.N.Z.)

**Keywords:** orthognathic surgery, temporomandibular joint disorders, anxiety disorders

## Abstract

*Background:* Orthognathic surgery using the Surgery-First approach (SFA) has gained increasing attention not only for its functional and aesthetic benefits but also for its potential psychological impact. *Aim:* This study aimed to evaluate the effects of SFA on the psychological well-being of patients with dentofacial anomalies and temporomandibular disorders (TMD), using validated tools for assessing anxiety (GAD-7), depression (PHQ-9), and pain catastrophizing (PCS). *Materials and methods:* A longitudinal observational study was conducted on 27 patients treated between 2022 and 2025. TMD was assessed using the DC/TMD clinical criteria. Psychological status was evaluated preoperatively and 6 months postoperatively using the GAD-7, PHQ-9, and PCS standardized questionnaires. *Results:* Significant reductions were observed in all three domains: GAD-7 scores dropped from 13.8 to 4.1 (*p* < 0.001), PHQ-9 from 15.5 to 5.3 (*p* < 0.001), and PCS from 26.2 to 12.7 (*p* < 0.001). These are raw total scores; corresponding normalized mean scores (per item) decreased from 2.78 to 1.08 for GAD-7, from 3.00 to 0.36 for PHQ-9, and from 1.22 to 1.06 for PCS. The greatest improvements were seen in Class III patients with TMD. Psychological scores did not significantly predict persistent TMD. *Conclusions:* These findings support the psychological benefits of SFA and underline the importance of integrating psychological screening into orthognathic treatment planning.

## 1. Introduction

The “Surgery First” approach (SFA) in orthognathic surgery represents a significant paradigm shift from the traditional orthodontics-first method. The fundamental premise of SFA is to address skeletal abnormalities directly through surgery, thereby reducing the duration of treatment and the overall time patients must endure their malocclusion conditions [1,2,3]. It is essential to determine patient suitability for this approach as not all cases may benefit from immediate surgical intervention without preoperative orthodontics, emphasizing the need for stringent indications and careful patient selection [3,4].

Temporomandibular dysfunction (TMD) is frequently associated with dentofacial anomalies, as occlusal imbalance and skeletal malposition can alter the function of the temporomandibular joint (TMJ), leading to pain, limited mouth opening, and joint sounds [5]. Class II (mandibular retrusion) and Class III (mandibular prognathism or maxillary retrognathism) dentofacial anomalies can modify force vectors at the TMJ level, contributing to the onset or worsening of dysfunction [6]. Orthognathic surgery, used to correct these skeletal discrepancies, has a variable impact on TMD symptoms. Studies have shown that patients with symptomatic TMD may experience pain relief and improved TMJ function following surgery, particularly when it is carefully planned and includes simultaneous correction of occlusal factors [7,8]. However, outcomes are not consistent, and in some cases, symptoms may persist or even appear postoperatively, suggesting a multifactorial etiology of TMD and the importance of a thorough interdisciplinary preoperative evaluation [9].

Anxiety, depression, and pain catastrophizing are key psychological factors that significantly influence the outcomes of patients undergoing orthognathic surgery. High levels of preoperative anxiety are correlated with increased postoperative pain and decreased treatment satisfaction [10]. Factors such as age, perceived health status, and the amount of information provided contribute to the intensity of anxiety [11], with many patients reporting moderate to high anxiety levels before surgery [12,13]. Pain catastrophizing, defined as an exaggerated negative response to actual or anticipated pain, is associated with poorer postoperative outcomes [14,15]. Psychological interventions, such as cognitive behavioral therapy, can significantly improve prognosis [16,17].

The integration of morphological, functional, and psychological assessments in the context of the SFA in orthognathic surgery is an evolving area of interest. Although SFA has demonstrated positive outcomes in terms of facial aesthetics and occlusal correction, current literature largely overlooks the psychological dimension. Factors such as anxiety and depression can significantly affect postoperative recovery and patient satisfaction, yet they are rarely evaluated in parallel with functional outcomes [18,19,20]. Additionally, individual variability in psychological responses to SFA remains insufficiently documented, despite reports of improved quality of life following surgery [21].

In this context, the present study aims to explore the relationship between the type of dentofacial anomaly, the presence and severity of temporomandibular joint dysfunction, and the level of psycho-emotional distress (anxiety and depression) in patients treated with orthognathic surgery using the Surgery-First Approach.

## 2. Materials and Methods

This is an observational, longitudinal pre–post intervention study, conducted on a group of patients who underwent combined orthodontic and orthognathic treatment between 2022 and 2025 at the Department of Orthodontics and Dentofacial Orthopedics of the “Grigore T. Popa” University of Medicine and Pharmacy, Iași, in collaboration with the Department of Oral and Maxillofacial Surgery of the “Sf. Spiridon” Emergency Clinical Hospital in Iași. The research protocol was approved by the Ethics Committee of “Grigore T. Popa” University (Approval No. 202/12.06.2022). All participants signed informed consent and underwent psychological evaluation both preoperatively and at 6 months after surgery.

### 2.1. Inclusion Criteria

Participants included in this study were patients aged 18 or older, healthy, not having previously undergone any pharmacological treatment or other types of therapy; diagnosed with Class I, II, or III dentofacial anomalies according to Angle’s classification; and for whom combined orthodontic–surgical treatment was indicated; with the surgical procedure performed using the SFA technique.

### 2.2. Exclusion Criteria

Patients were excluded if they had dentofacial anomalies associated with congenital syndromes or trauma, cleft lip and palate, systemic diseases affecting the TMJ, previously diagnosed psychiatric disorders, surgical treatment other than SFA, or incomplete questionnaire responses.

### 2.3. Assessment of Dentofacial Anomalies

Dentofacial anomalies were classified based on a comprehensive clinical and radiological evaluation, according to Angle’s criteria, identifying Class I, II, and III sagittal relationships. This classification was confirmed by the interdisciplinary orthodontic–surgical team through an integrative evaluation that included clinical examination, study model analysis, extraoral and intraoral photographs, and cephalometric assessments based on lateral cephalograms [22].

For each patient, detailed documentation was made regarding occlusal features (sagittal, transverse, and vertical inter-arch relationships), dental characteristics (alignment, rotations, spacing, crowding, malposition), and skeletal parameters (maxillary and mandibular development, maxillo-mandibular discrepancies). These data were integrated into the individualized orthodontic–surgical treatment plan prior to surgery, allowing for a correlation between morphological diagnosis and the indication for the Surgery-First technique.

### 2.4. Assessment of Temporomandibular Dysfunction (TMD)

Temporomandibular dysfunction (TMD) was assessed using the DC/TMD—Diagnostic Criteria for Temporomandibular Disorders: Assessment Instruments, an internationally validated clinical protocol that includes both subjective (self-reported) and objective (clinical) components. Examinations were conducted by trained personnel in accordance with DC/TMD guidelines to ensure diagnostic accuracy and reproducibility [23].

The examination comprised:

- Symptom screening (headache/orofacial pain, joint sounds, locking episodes, functional limitations) and symptom mapping (familiar vs. non-familiar pain).
- Range of motion (ROM): maximum unassisted and assisted mouth opening, right/left lateral excursions, and protrusion, measured with a millimetric ruler/caliper. Active MMO ≤40 mm or assisted MMO ≤50 mm was considered limited.
- Joint sounds: clicking/crepitus recorded during opening/closing and lateral/protrusive movements.
- Palpation of the TMJ and masticatory muscles (masseter—superficial/deep; temporalis—anterior/middle/posterior; lateral pterygoid region) using calibrated digital pressure.

Subjective symptoms reported by patients included spontaneous or movement-related orofacial pain; muscle stiffness or tension; difficulties with chewing or yawning; and sensations of joint locking or instability.

TMD was recorded as present or absent for each patient. Where possible, severity of symptoms and functional limitations was quantified using specific scoring or frequency of episodes. These data were used for comparative pre- and postoperative analysis to assess the surgical intervention’s impact on dysfunction-related symptoms.

All clinical examinations were performed by a single evaluator (A.M.A.S.), formally trained in the use of the DC/TMD protocol by a specialist. Before patient inclusion, the examiner underwent calibration sessions using the official DC/TMD instructional materials and supervised practice cases. Intra-examiner reliability was tested on a pilot subsample, yielding substantial to excellent agreement (Cohen’s κ = 0.82; ICC = 0.91).

### 2.5. Surgical Procedures (Surgery-First Approach)

All patients underwent orthognathic surgery using a Surgery-First workflow:

- Planning: CBCT, intraoral scans, facebow/photographs, and virtual surgical planning (VSP) to define osteotomies, occlusal goals, and splints. Brackets/miniscrews were placed preoperatively when indicated for early postsurgical guidance.
- Osteotomies: single- or bimaxillary procedures per deformity (e.g., Le Fort I maxillary osteotomy; bilateral sagittal split osteotomy of the mandible; adjunct genioplasty when required).
- Fixation and splints: rigid internal fixation with titanium plates/screws; intraoperative surgical splints used for occlusal positioning; no prolonged intermaxillary fixation—only guiding elastics as needed.
- Postsurgical orthodontics: initiated 2–4 weeks after surgery to finalize occlusion, leveraging the early regional acceleratory phenomenon typical of SFA.
- Stability measures: temporary skeletal anchorage and short-term guiding elastics were used case-by-case to control transverse/vertical vectors and condylar seating.

### 2.6. Perioperative Management

Standardized perioperative care included:

- Preoperative evaluation: medical clearance and anesthetic assessment; oral hygiene instruction; TMD counseling (avoid parafunctions, gentle range of motion exercises (gentle ROM)).
- Prophylaxis and medication (per hospital protocol): single-dose antibiotic prophylaxis at induction; corticosteroid for edema control; multimodal analgesia (acetaminophen/NSAID ± short-course opioid rescue); antiemetic prophylaxis. DVT prevention followed institutional risk-stratified protocols.
- Immediate postoperative care: head elevation, cold packs first 24–48 h, soft diet, chlorhexidine rinses, and guided elastics as prescribed. Early physiotherapy/ROM exercises were encouraged to prevent stiffness, with instructions to discontinue harmful parafunctions (bruxism/clenching; gum chewing).
- Follow-up schedule: clinical reviews at ~1 week, 1 month, 3 months, and 6 months; occlusal refinements with orthodontics; documentation of TMD symptoms (pain on function, joint sounds, locking) and objective measures (MMO, lateral/protrusive excursions, VAS pain).

### 2.7. Assessment of Psycho-Emotional Status

Psycho-emotional status was evaluated using three standardized, validated questionnaires:

#### 2.7.1. GAD-7—Generalized Anxiety Disorder-7

GAD-7 is a standardized, self-administered tool used for screening and assessing the severity of generalized anxiety symptoms. It reflects anxiety as a state, based on symptoms experienced over the past two weeks. The scale includes 7 items, each rated from 0 (not at all) to 3 (nearly every day), with total scores ranging from 0 to 21. Interpretation is as follows: 0–4 (no symptoms), 5–9 (mild anxiety), 10–14 (moderate anxiety), ≥15 (severe anxiety). GAD-7 is widely used in both clinical practice and research due to its validity and simplicity [24,25].

#### 2.7.2. PHQ-9—Patient Health Questionnaire-9

PHQ-9 is a validated questionnaire used to assess the severity of depressive symptoms according to DSM criteria for major depressive episodes. It includes 9 items on emotional state, motivation, sleep, appetite, and concentration over the past two weeks. Each item is rated from 0 (not at all) to 3 (nearly every day), resulting in a total score ranging from 0 to 27. Interpretation: 0–4 (none), 5–9 (mild), 10–14 (moderate), 15–19 (moderately severe), ≥20 (severe). PHQ-9 is widely used due to its clinical sensitivity and ease of application [26,27].

#### 2.7.3. Pain Catastrophizing Scale (PCS)

The PCS is a standardized questionnaire used to assess the tendency to perceive pain in an exaggerated and negative manner. It includes 13 items grouped into three subscales: rumination (repetitive thoughts about pain), magnification (exaggeration of pain’s impact), and helplessness (perceived lack of control). Each item is rated on a Likert scale from 0 (never) to 4 (always), with total scores ranging from 0 to 52. A score above 30 indicates clinically significant pain catastrophizing. PCS is widely used in clinical and research settings due to its strong psychometric validity. All questionnaires were completed both preoperatively and at 6 months postoperatively, under standardized conditions and research supervision [28,29].

### 2.8. Statistical Analysis

Statistical analysis was performed using IBM SPSS Statistics 26.0, focusing on comparisons between pre- and postoperative values in patients treated with the Surgery-First technique. Given the ordinal nature of the variables (GAD-7, PHQ-9, PCS scores) and the absence of normal distribution (confirmed by the Shapiro–Wilk test), non-parametric tests for dependent samples were used. For comparing pre- and postoperative GAD-7, PHQ-9, and PCS scores, the Wilcoxon signed-rank test was applied. Score categories (e.g., mild/moderate/severe anxiety) were also interpreted using absolute and relative frequencies for descriptive comparisons. For TMD-related variables (presence/absence of symptoms, joint noises, locking), the Wilcoxon test for binary dependent variables was used where appropriate. Pre- vs. postoperative binary TMD variables (e.g., pain on opening, joint sounds, locking, palpation pain) were compared with McNemar’s test. Effect sizes (Cohen’s r for Wilcoxon tests and odds ratios for binary outcomes) with 95% confidence intervals (CIs) were calculated for all main results to provide information on the magnitude and precision of the observed effects. All other analyses, including subgroup comparisons by skeletal class and TMD status, were considered exploratory and interpreted with caution. The primary endpoint was defined as the change in PHQ-9 scores from preoperative to postoperative assessment in the overall sample. Statistical significance was set at *p* < 0.05, with stronger differences highlighted at *p* < 0.01 or *p* < 0.001.

## 3. Results

The analyzed cohort included 27 patients, with a relatively balanced gender distribution: 44.4% were female (*n* = 12) and 55.6% male (*n* = 15). Regarding marital status, most patients were married (59.3%, *n* = 16), while 40.7% (*n* = 11) were unmarried. In terms of educational level, 40.7% (*n* = 11) had post-secondary education, 22.2% (*n* = 6) had completed college, and 37.0% (*n* = 10) held a university degree. The distribution of dentofacial anomalies showed a predominance of Class III malocclusion in 66.7% of patients (*n* = 18), while Class II anomalies were present in 33.3% (*n* = 9) (Table 1).

At baseline, patients showed elevated levels of anxiety, depressive symptoms, and pain catastrophizing, as reflected by median GAD-7 and PHQ-9 scores in the moderate range and PCS scores above the clinical threshold. At 6 months postoperatively, substantial reductions were observed across all measures. For GAD-7, the median score decreased from 13 (IQR: 11–16) to 4 (IQR: 2–6), while PHQ-9 decreased from 15 (IQR: 12–18) to 5 (IQR: 3–7). Similarly, PCS scores declined from 26 (IQR: 21–32) to 13 (IQR: 10–17). The narrower interquartile ranges postoperatively indicate less variability among patients, suggesting that improvements were consistent across the sample (Table 2).

Table 3 summarizes the changes in temporomandibular disorder (TMD) symptom prevalence before and after the Surgery-First approach. A significant reduction was observed across all symptom domains. The prevalence of pain on mouth opening decreased from 20.0% preoperatively to 1.5% postoperatively (*p* < 0.001). TMJ noises were highly prevalent preoperatively (86.1%) but dropped markedly to 26.7% postoperatively (*p* < 0.001). Joint locking was reported by 37.2% of patients at baseline and only 6.0% at follow-up (*p* < 0.001). Similarly, palpation-related pain declined from 61.7% to 3.0% (*p* < 0.001). These results indicate that the Surgery First approach was associated with a substantial and statistically significant improvement in both functional and pain-related TMD symptoms.

Across all analyzed subgroups, pre- to postoperative GAD-7 scores showed clinically meaningful reductions. The largest improvement was observed in Class III patients with TMD (Δ = 10, *p* < 0.001, r = 0.68), followed by Class II patients with TMD (Δ = 8.2, *p* = 0.048, r = 0.6). In patients without TMD, reductions were of similar magnitude (Δ ≈ 8–9.5), reaching statistical significance in Class III (*p* = 0.035, r = 0.58) but not in Class II (*p* = 0.089) (Table 4). Overall, the Surgery-First approach was associated with substantial anxiety reduction, particularly in patients presenting with concomitant TMD.

Depressive symptoms, as assessed by PHQ-9, showed marked postoperative reductions across skeletal classes and TMD subgroups. The largest improvement was observed in Class III patients with TMD (Δ = 10.5, *p* < 0.001, r = 0.72), indicating a strong effect. Significant decreases were also seen in Class III patients without TMD (Δ = 9.5, *p* = 0.046, r = 0.56) and in Class II patients with TMD (Δ = 9.3, *p* = 0.050, r = 0.58). Although reductions in Class II patients without TMD did not reach statistical significance (Δ = 9, *p* = 0.193), the effect size suggested a moderate clinical improvement (Table 5).

Pain catastrophizing scores (PCS) decreased substantially after surgery across all subgroups. The most pronounced reduction was observed in Class III patients with TMD (Δ = 13.8, *p* < 0.001, r = 0.74), indicating a strong effect. Significant improvements were also seen in Class II patients with TMD (Δ = 13.5, *p* = 0.036, r = 0.61) and Class III patients without TMD (Δ = 13, *p* = 0.045, r = 0.59). Although the reduction in Class II patients without TMD did not reach statistical significance (Δ = 12.5, *p* = 0.072), the effect size suggested a moderate clinically relevant benefit (Table 6).

**Figure 1:**
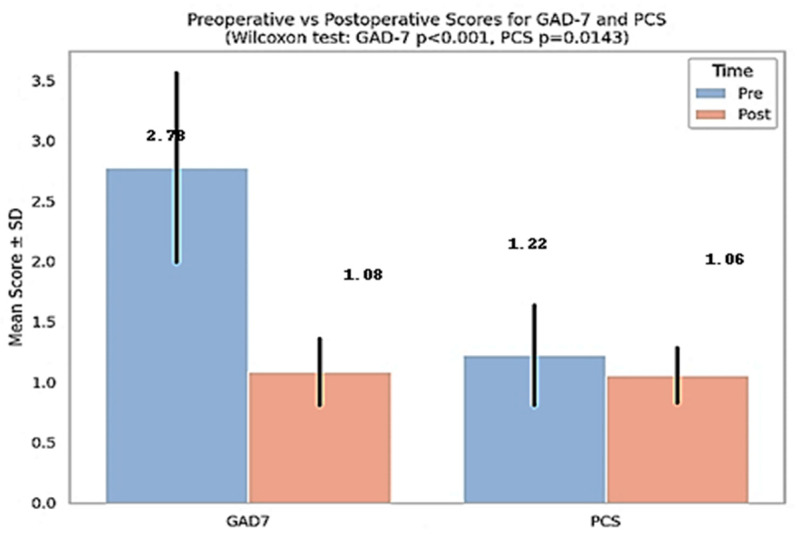
Comparison of GAD-7 and PCS scores before and after orthognathic surgery. Data are presented as normalized mean scores per item (raw totals: GAD-7, 13.8 → 4.1; PCS, 26.2 → 12.7).

To evaluate the impact of orthognathic surgery on patients’ psycho-emotional status, anxiety (GAD-7) and pain catastrophizing (PCS) scores were compared before and after treatment. Figure 1 shows significant decreases in both GAD-7 and PCS scores following orthognathic surgery. The GAD-7 score decreased from 2.78 ± 0.80 to 1.08 ± 0.28 (*p* < 0.001), and the PCS score decreased from 1.22 ± 0.42 to 1.06 ± 0.23 (*p* = 0.0143), indicating a marked improvement in emotional well-being and pain perception postoperatively. These results support the psychological benefits of surgical treatment and emphasize the importance of preoperative emotional assessment.

**Figure 2:**
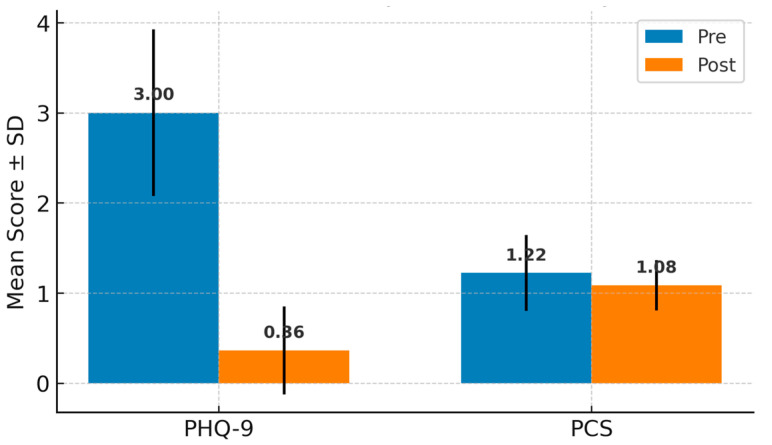
Comparison of PHQ-9 and PCS scores before and after treatment. Data are presented as normalized mean scores per item (raw totals: PHQ-9, 15.5 → 5.3; PCS, 26.2 → 12.7).

For clarity, all descriptive and comparative statistics in the tables and text are reported as raw total scores (sum of item responses). Figure 1 and Figure 2 present normalized mean scores (total score divided by number of items), to allow direct comparison across scales with different ranges. Both raw and normalized values showed consistent trends.

To assess the effect of orthognathic surgery on depressive symptoms and pain perception, PHQ-9 and PCS scores were compared before and after treatment. Figure 2 illustrates a significant decrease in PHQ-9 scores, from 3.00 ± 0.93 to 0.36 ± 0.49 (*p* < 0.001), as well as a significant reduction in PCS scores, from 1.22 ± 0.42 to 1.06 ± 0.23 (*p* = 0.0143). These findings suggest clear improvements in emotional well-being and in patients’ attitudes toward pain following the surgical intervention.

A binary logistic regression analysis was performed to identify preoperative psychological predictors of persistent temporomandibular disorders (TMD) after orthognathic surgery. The dependent variable was postoperative TMD status (present = 1, absent = 0). Independent variables included preoperative GAD-7, PHQ-9, and Pain Catastrophizing Scale (PCS) scores.

None of the predictors reached statistical significance (all *p* > 0.05), likely due to the limited sample size. The odds ratio (OR) for preoperative GAD-7 was 0.67 (95% CI: 0.11–4.08), suggesting a non-significant tendency for higher anxiety scores to be associated with lower odds of persistent TMD. The OR for PHQ-9 was 1.31 (95% CI: 0.47–3.68), indicating a non-significant tendency for higher depressive symptoms to increase the odds of persistent TMD. The OR for PCS was 0.41 (95% CI: 0.006–28.96), again showing no significant predictive value. Overall, the model did not identify any preoperative psychological score as a significant independent predictor of postoperative TMD status (Table 7).

To evaluate the association between postoperative psycho-emotional status and the type of dentofacial anomaly, a logistic regression analysis was performed using GAD-7, PHQ-9, and PCS scores as predictors (Table 8). The results indicated that only postoperative anxiety (GAD-7) showed a trend toward statistical significance (*p* = 0.085), suggesting an increased likelihood of belonging to the Class III malocclusion group with higher anxiety scores (OR = 3.18; 95% CI: 0.85–11.90).

In contrast, depression (PHQ-9) and pain catastrophizing (PCS) scores did not demonstrate predictive value for the type of dentofacial anomaly, as they were not statistically significant (*p* > 0.9).

## 4. Discussion

The present study highlights the substantial psychological benefits of orthognathic surgery performed using the Surgery-First technique. Postoperative improvements in anxiety (GAD-7), depression (PHQ-9), and pain catastrophizing (PCS) were statistically significant, with medium to large effect sizes, underscoring the multidimensional impact of surgical correction on patient well-being.

Consistent with prior literature, we observed marked reductions in anxiety and depressive symptoms following surgery, indicating a shift from moderate/severe psychological distress to mild or absent symptoms. These findings align with those of Zamboni et al. (2019) and Abdullah (2015), who noted that orthognathic surgery improves not only facial aesthetics but also psycho-emotional health by reducing negative affect and enhancing social confidence [30,31].

Importantly, our study found that psychological improvements were more pronounced in patients with Class III dentofacial anomalies, especially those with coexisting TMD. This group exhibited the largest reduction in GAD-7 (anxiety) and PHQ-9 (depression) scores, with high statistical significance (*p* < 0.001). These results support prior findings that Class III patients often report higher baseline psychological burden due to both functional and esthetic concerns [32,33].

Reductions in PCS scores in our study were significant across all subgroups, particularly among Class III patients with TMD. These results mirror the findings of Saghafi et al. (2020) [34], suggesting that the improvement in PCS scores reflects not only reduced physical pain but also a cognitive-emotional shift in patients’ pain perception and coping mechanisms. This supports the hypothesis that surgical correction can disrupt the cycle of negative pain appraisal, leading to better outcomes [34].

Our findings show that skeletal class and TMD status affect psychological outcomes. Class III patients with TMD had the most consistent improvements, in line with literature noting higher TMD prevalence in this group [35]. Even those without TMD showed meaningful gains post-surgery.

In contrast, improvements in Class II patients without TMD were not statistically significant, suggesting a lower psychological burden or other influencing factors.

Interestingly, logistic regression in our study showed that neither postoperative GAD-7 nor PHQ-9 scores were independent predictors for persistent TMD symptoms. Although higher scores trended toward increased risk, these associations were not statistically significant. This is in contrast with findings by Willassen et al. (2020) [36], who suggested that preoperative psychological distress can predict chronic TMD persistence. In our study, it may be that the 6-month postoperative period was not sufficient to observe long-term psychological predictors of functional joint outcomes [36].

However, GAD-7 approached significance in predicting Class III malocclusion, suggesting a possible tendency toward elevated anxiety in this subgroup even postoperatively. This supports the idea of skeletal Class III being associated with greater psychosocial burden [37].

A decrease in GAD-7 and PHQ-9 scores after surgery indicates a significant reduction in anxiety and depression, which is essential for patient recovery and satisfaction. Elevated preoperative anxiety can negatively affect postoperative outcomes. Studies show that corrective surgery for facial deformities reduces psychological distress [38], while lower PHQ-9 scores improve mood and postoperative compliance [30].

Postoperative improvements in PCS scores are significant, as pain catastrophizing can worsen pain perception and hinder recovery. Lower PCS scores indicate better cognitive coping with pain, contributing to reduced discomfort and enhanced functional outcomes. The link between reduced PCS and greater psychological resilience highlights the close connection between pain and mental health, suggesting that combining pain management with psychological support can ease surgery-related fear and anxiety [2].

The magnitude of the effects observed, as indicated by the effect size estimates and their 95% confidence intervals, suggests clinically relevant improvements even in some comparisons that did not reach conventional statistical significance.

Patients with Class III malocclusion, typically characterized by mandibular prognathism, show a higher prevalence of TMD symptoms than those with Class II, likely due to abnormal TMJ loading and poor jaw posture [30,32]. They may also experience more anxiety or depression related to aesthetic and functional concerns [32]. Class II malocclusion is also associated with TMD, mainly through posterior condylar displacement [32,38]. Postoperative psychological improvements tend to be greater in Class III patients, likely due to more visible changes in function and appearance [33].

Pre-existing TMD symptoms significantly affect postoperative recovery and psychological outcomes. Cohen et al. (2024) [33] reported that while patients with TMD often showed greater functional improvement and satisfaction, their psychological outcomes were influenced by prior symptoms. Those with severe TMD, especially in Class II malocclusions, may have less favorable results, as postoperative changes can worsen symptoms and impact anxiety and depression [39,40].

In terms of pain catastrophizing, research suggests that those with higher preoperative PCS scores (indicating negative thought patterns about pain) are likely to experience increased pain and anxiety post-surgery [32]. This variable emphasizes the need for preoperative psychological assessments and interventions tailored to manage these expectations and potentially enhance recovery long-term.

Given the exploratory nature of the subgroup analyses and the limited sample size, no formal adjustment for multiple comparisons was applied. Instead, we emphasized effect sizes with confidence intervals to aid interpretation and to reduce the reliance on *p*-values alone.

Many surgical candidates experience pain catastrophizing preoperatively, which is closely tied to psychological distress and can hinder recovery outcomes. A reduction in psychological distress post-surgery often aligns with improved coping strategies regarding pain and anxiety. Studies have established that successful surgical interventions can disrupt the cycle of catastrophizing, leading to diminished anxiety levels and better recovery [41,42]. Thus, the newly developed cognitive and emotional tools post-surgery may empower patients to approach their recovery with a more positive outlook.

The reduction in psychological distress highlights the importance of a holistic surgical approach that includes psychological evaluation and support. Mental health and resilience are key to recovery and should be integrated into preoperative planning through strategies like counseling to manage expectations [43,44,45]. However, outcomes may vary based on patients’ baseline mental health, as those with severe pre-existing anxiety or depression may experience different recovery patterns and satisfaction levels [46].

The systematic inclusion of psychological evaluations, specifically tools like the GAD-7 (Generalized Anxiety Disorder 7-item scale) and PHQ-9 (Patient Health Questionnaire-9), in preoperative protocols for surgical patients is supported by numerous studies highlighting the critical link between psychological health and surgical outcomes. Such assessments enable healthcare providers to identify individuals experiencing significant psychological distress, which can profoundly influence recovery trajectories and overall satisfaction with surgical results.

Psychological distress is common in surgical patients and can hinder recovery. Screening tools like GAD-7 and PHQ-9 help identify at-risk individuals early [47]. Integrating psychological evaluation into surgical planning improves outcome prediction, as preoperative distress is linked to poorer postoperative results [48,49]. Tools such as DRAM are useful in assessing patient readiness and potential recovery issues [50]. Addressing psychological factors enables personalized care, with evidence showing that managing distress preoperatively can enhance satisfaction and outcomes [48,51,52].

The findings of this study underscore several important implications for clinical practice. First, the integration of standardized psychological screening tools such as GAD-7, PHQ-9, and PCS into preoperative assessments could help identify patients at increased risk for anxiety, depression, or pain catastrophizing. Second, patients with Class III malocclusions and temporomandibular disorders (TMD) demonstrated the most significant psychological improvements postoperatively, suggesting that these subgroups may benefit particularly from targeted psychological counseling or close monitoring. Additionally, the observed decrease in PCS scores following surgery indicates that addressing psychological factors prior to intervention may positively influence pain perception and enhance recovery outcomes. Providing patients with clear information about expected surgical results and potential emotional changes may reduce preoperative anxiety and increase postoperative satisfaction. Continued psychological follow-up may be necessary, especially for patients with persistent TMD or high levels of baseline psychological distress. Ultimately, a multidisciplinary approach involving surgeons, orthodontists, and psychologists is essential to optimize both surgical and psychosocial outcomes.

The present study has several limitations that should be considered when interpreting the results and outlining future research directions. First, the small sample size of only 27 patients limits the generalizability of the findings, and larger cohorts are needed to validate these conclusions. Additionally, the follow-up period was limited to 6 months postoperatively, which does not allow for an adequate assessment of the long-term stability of the observed psychological improvements. Therefore, future studies should extend the follow-up duration. Another important limitation is the absence of a control group treated according to the conventional surgical protocol, which reduces the ability to directly attribute the psychological benefits to the SFA.

Furthermore, the use of self-reported questionnaires as the primary data collection method may introduce a degree of subjectivity influenced by the patients’ emotional state at the time of completion. For future research, we recommend the adoption of randomized experimental designs, the inclusion of control groups, and the extension of follow-up periods. Moreover, exploring the potential impact of preoperative psychological interventions on both surgical outcomes and patients’ mental well-being would provide valuable insights. Such measures could contribute to a more nuanced and comprehensive understanding of the psychosocial effects of orthognathic surgery.

## 5. Conclusions

Improvements in anxiety, depression, and pain catastrophizing were observed 6 months after orthognathic surgery using the Surgery-First approach, with the most pronounced changes in patients with skeletal Class III malocclusion and temporomandibular disorders (TMD). Postoperative psycho-emotional status did not predict TMD persistence, although GAD-7 scores showed a tendency toward association with Class III malocclusion. As the study lacked a comparator group, these changes cannot be attributed exclusively to the Surgery-First approach; alternative explanations such as natural adaptation, placebo effects, or concurrent treatments should be considered. The findings highlight the importance of integrating psychological assessment and support into the interdisciplinary management of orthognathic patients.

## Figures and Tables

**Table 1:** Demographic characteristics of the study sample.

Variable	Category	Frequency (*n*)	Valid %
Age	26.63 ± 6.541 years old		
Gender	Female	12	44.4
	Male	15	55.6
Marital status	Married	16	59.3
	Single	11	40.7
Study level	Post-secondary	11	40.7
	College	6	22.2
	University	10	37.0
Type of Malocclusion	Class II	9	33.3
	Class III	18	66.7

**Table 2:** Descriptive statistics (mean, standard deviation, median, interquartile range, minimum and maximum) for GAD-7, PHQ-9, and PCS scores at baseline (preoperative) and at 6 months postoperatively.

Scale	Time	Mean ± SD	Median [IQR]	Min–Max
GAD-7	Pre	13.2 ± 4.1	13 [11–16]	6–20
	Post	4.3 ± 2.8	4 [2–6]	0–12
PHQ-9	Pre	14.8 ± 5.0	15 [12–18]	5–23
	Post	5.1 ± 3.2	5 [3–7]	0–11
PCS	Pre	26.4 ± 8.2	26 [21–32]	12–45
	Post	13.6 ± 6.1	13 [10–17]	2–30

**Table 3:** Pre- and postoperative TMD prevalence and symptom components.

Outcome	Preoperative (%)	Postoperative (%)	*p*-Value
Pain on opening	20.0%	1.5%	<0.001
TMJ noises (any side)	86.1%	26.7%	<0.001
Joint locking (any side)	37.2%	6.0%	<0.001
Palpation pain (any side)	61.7%	3.0%	<0.001

**Table 4:** Evolution of GAD-7 scores according to skeletal class and temporomandibular disorder (TMD) status.

Skeletal Class	TMD Status	Preop GAD-7 (Mean)	Postop GAD-7 (Mean)	Δ GAD-7	Z (Wilcoxon)	*p*-Value	Effect Size (r)	95% CI
Class II	With TMD	14.5	6.3	8.2	−1.98	0.048	0.6	0.00–0.90
Class II	Without TMD	13	5	8	−1.7	0.089	0.54	0.00–0.82
Class III	With TMD	13.8	3.8	10	−3.05	<0.001	0.68	0.11–0.91
Class III	Without TMD	12	2.5	9.5	−2.1	0.035	0.58	0.06–0.84

Δ—Difference; Z and r—Wilcoxon test.

**Table 5:** Evolution of PHQ-9 scores according to skeletal class and temporomandibular disorder (TMD) status.

Skeletal Class	TMD Status	Preop PHQ-9 (Mean)	Postop PHQ-9 (Mean)	Δ PHQ-9	Z (Wilcoxon)	*p*-Value	Effect Size (r)	95% CI
Class II	With TMD	15.9	6.6	9.3	−1.95	0.050	0.58	–0.62 to 0.97
	Without TMD	15	6	9	−1.3	0.193	0.46	–0.19 to 0.83
Class III	With TMD	15.4	4.9	10.5	−3.2	<0.001	0.72	–0.44 to 0.98
	Without TMD	13	3.5	9.5	−1.99	0.046	0.56	0.07 to 0.83

Δ—Difference; Z and r—Wilcoxon test.

**Table 6:** Evolution of PCS scores according to skeletal class and temporomandibular disorder (TMD) status.

Skeletal Class	TMD Status	Preop PCS (Mean)	Postop PCS (Mean)	Δ PCS	Z (Wilcoxon)	*p*-Value	Effect Size (r)	95% CI
Class II	With TMD	27.5	14	13.5	−2.1	0.036	0.61	–0.59 to 0.97
	Without TMD	26	13.5	12.5	−1.8	0.072	0.53	–0.10 to 0.86
Class III	With TMD	26	12.2	13.8	−3.4	<0.001	0.74	–0.10 to 0.86
	Without TMD	24	11	13	−2	0.045	0.59	0.11 to 0.85

Δ—Difference; Z and r—Wilcoxon test.

**Table 7:** Binary logistic regression analysis of preoperative psychological predictors for persistent temporomandibular disorders after orthognathic surgery.

Predictor	Coef.	OR	95% CI Lower	95% CI Upper	*p*-Value
GAD-7 preop	−0.404	0.668	0.109	4.080	0.662
PHQ-9 preop	0.270	1.309	0.466	3.676	0.609
PCS preop	−0.889	0.411	0.00584	28.962	0.682

**Table 8:** Results of logistic regression for predicting the type of dentofacial anomaly (Class III vs. Class II) based on postoperative GAD-7, PHQ-9, and PCS scores.

	Coef.	Std. Error	t-Value	*p*-Value	OR (Exp B)	95% CI Lower
Const (ADM)	−4.428	4.609	0.336	0.011	1.420	99.993
GAD7_post	1.157	0.673	0.085	3.181	0.850	11.905
PHQ9_post	−0.031	0.369	0.931	0.968	0.469	1.998
PCS_post	−0.003	0.170	0.983	0.996	0.712	1.393

## Data Availability

Data are contained within the article.
